# Can apelin play a role in the etiology of tinnitus?

**DOI:** 10.3906/sag-1812-11

**Published:** 2019-06-18

**Authors:** Nuray ENSARİ, Özer Erdem GÜR, Nilgün GÜR, Ömer Tarık SELÇUK, Levent RENDA, Mustafa Deniz YILMAZ, Mehmet Türker ÖZTÜRK, Yeşim ÇEKİN

**Affiliations:** 1 University of Health Sciences Antalya Training and Research Hospital, Ear, Nose, and Throat Clinic, Antalya Turkey2; 2 University of Health Sciences Antalya Training and Research Hospital, Microbiology Clinic, Antalya Turkey

**Keywords:** Apelin, oxidative stress, tinnitus

## Abstract

**Background/aim:**

Tinnitus is seen in 15% of the general population; in 1%–6% of this number, the quality of life is seriously affected by this chronic condition. Chemical, oxidative, and emotional stressors are important in terms of the clinical course of tinnitus. Apelin is an endogenous peptide which is an oxidative stress mediator. It has been shown that the apelin/APJ (apelin junction receptor) system plays various roles in the physiology and pathophysiology of many organs. However, the role of the apelin/APJ system as an oxidative stress mediator in tinnitus is unknown. We investigated the level of apelin in patients with normal hearing and bilateral tinnitus.

**Materials and methods:**

We enrolled patients diagnosed with bilateral idiopathic tinnitus. Tinnitus severity was determined using the tinnitus handicap inventory (THI). We recorded the levels of plasma apelin-13 and biochemical parameters.

**Results:**

The mean apelin level of the control group was higher than that of the patient group (P = 0.002). A significant negative correlation was evident between the apelin level and the THI (r = –0.460, P = 0.003). The triglyceride (TG) level was significantly higher in the patient group than in the control group (P < 0.001).

**Conclusions:**

In our study, we found a negative correlation between apelin and tinnitus severity. Thus, apelin may play a role in the pathophysiology of idiopathic tinnitus, and may be prescribed during follow-up to reduce oxidative stress in the future. Further clinical studies on the effects of the apelin/APJ (apelin junction receptor) system and the effects of antioxidants in patients with inflammatory diseases are required.

## 1. Introduction

Tinnitus, which affects 15% of the general population, is the perception of imagined sound in the absence of an external stimulus (1); the condition is most prevalent in males aged 40–70 years. Tinnitus alone is not a clinical sign; tinnitus is a symptom of several diseases. Tinnitus becomes chronic in 1%–6% of patients and seriously reduces quality of life. In addition, tinnitus-related factors such as hearing loss, hypertension, hormonal disorders, anxiety, depression, sleep disorders, and concentration problems are more often encountered than tinnitus per se (2). Nowadays, etiopathogenesis is still unclear in 50%–60% of patients who have tinnitus (1). Hearing loss, acoustic trauma, Meniere’s disease, vestibular schwannoma, temporomandibular joint disease, and the use of otoacoustic drugs trigger tinnitus associated with impaired otological functioning. In addition, metabolic, neurogenic, or psychogenic disorders, environmental factors, stress, or obesity may cause tinnitus (3). 

Stress is important in terms of the clinical course of tinnitus. Hearing is particularly sensitive to chemical, oxidative, and emotional stressors (4). Apelin (a hormone) mediates oxidative stress, and is expressed by the vascular endothelium and adipose tissue. The aim of this study was to investigate the apelin mediator as a new oxidative stress parameter in tinnitus patients. 

In 1993, O’Down et al. discovered a gene encoding a 380-amino acid protein similar in sequence to the angiotensin II type I receptor gene (5). The gene was termed APJ (the apelin receptor) and, in 1998, Tatemato et al. showed that apelin was the endogenous ligand (6). APJ is a member of the G-protein-binding receptor family, all members of which have 7 transmembrane regions. APJ is encoded in the Xq25-26.1 region. A preproapelin protein of 77 amino acids is initially synthesized and then fragmented into peptides with varying numbers of amino acids (e.g., apelin-10, -11, -12, -13, -15, -17, -19, and -36). Apelin-13 is the primary active isoform, binding specifically to APJ (7). 

Apelin vasoconstricts damaged vessels, and thus serves as an indicator of the severity of coronary artery stenosis (8). If apelin, which is abundant in vessel endothelium, plays a role during oxidative ear stress, and if tinnitus were affected by apelin, apelin might exert protective and therapeutic effects. 

## 2. Materials and methods 

The study was approved by the Clinical Research Ethics Committee of Antalya Training and Research Hospital. Informed consent was obtained from all patients and controls. No subject had diabetes, dyslipidemia, or cardiovascular disease. We included 40 patients aged >18 years with bilateral idiopathic tinnitus, no ear pathology, and normal hearing. Tinnitus severity was determined using the THI; the scores range from 0–16 points: mild (only evident in a silent environment), 18–36: fair (easily masked by surrounding noise and easily forgotten during activity), 38–56: moderate (despite awareness of background noise, daily activities can still be performed), 58–76: severe (present almost always, disrupting sleep and daily activities), and 78–100: catastrophic (present always, disrupting sleep and daily activities). The control group contained 40 healthy volunteers aged >18 years with normal ears and no tinnitus.

For all subjects, 10-mL amounts of fasting venous blood were collected and plasma apelin levels assayed via ELISA. Blood samples were immediately centrifuged for 20 min at 4000 rpm and stored at –80 °C. We used a human AP 13 ELISA kit (range: 0.77–200 pg/mL, sensitivity: 0.658 pg/mL) (Sunred Biotechnology, Shanghai, China) and a fully automatic MicroELISA device (Triturus/Grifols, Barcelona, Spain). We also measured the levels of fasting glucose (GLU), alanine aminotransferase (ALT), aspartate aminotransferase (AST), blood urea nitrogen (BUN), creatinine, triglycerides (TG), low-density lipoprotein (LDL), high-density lipoprotein (HDL), total cholesterol, hemoglobin (Hb), and hemoglobin A1c (Hb A1c) employing the Beckman–Coulter au5800 and LH 780 platforms. 

### 2.1. Statistical analysis

Statistical analyses were performed with the aid of IBM SPSS Statistics for Windows version 23.0 (IBM Corp., Armonk, NY, USA). Categorical variables were compared via Pearson chi-square testing. The normalities of between-group differences were assessed using the Shapiro–Wilk test. The Mann–Whitney U-test and Student’s t-test were employed to analyze nonnormally and normally distributed numerical data, respectively. Spearman rank correlation or Pearson correlation analyses were used to explore the association of apelin levels with various biochemical factors and the THI. The results are expressed as numbers (n) with percentages (%), means ±standard deviations (SDs), or medians (minima minus maxima). A P-value <0.05 was considered to reflect statistical significance.

## 3. Results

Gender distribution did not differ significantly between patients and controls (P = 0.478). No significant between-group difference was evident in any biochemical parameter (aSpearman rank correlation, bPearson correlation) (all P > 0.05).

**Figure 1:**
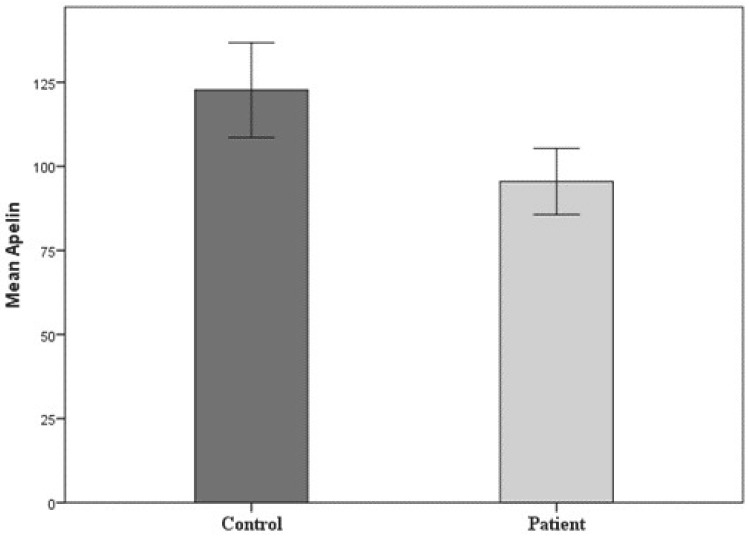
The apelin levels of the 2 groups.

**Figure 2:**
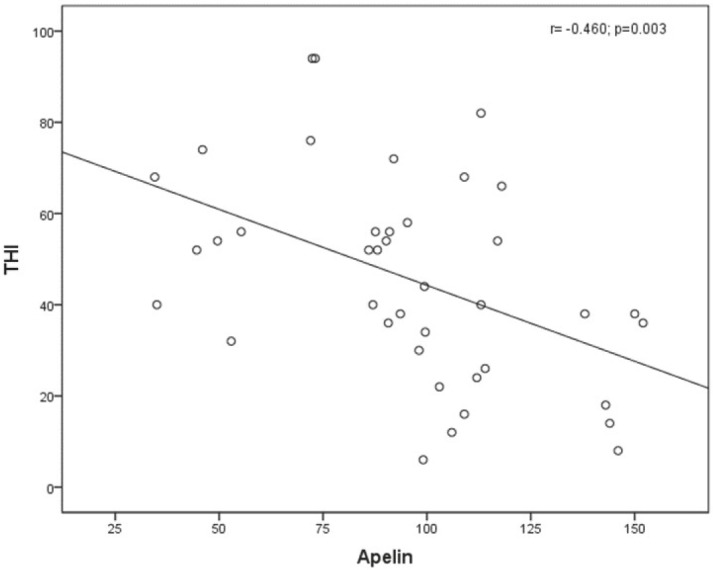
Correlations between the apelin levels and the THIs.

 The comparisons between apelin levels and biochemical parameters in both groups are shown in the Table. In the subgroup analysis, the serum apelin levels of the tinnitus patients were significantly lower than those of the control group (96.7 [34.5–152] pg/mL vs 122.6 [52.1–236] pg/mL; P = 0.002) (Figure 1), as was the mean HDL level (P = 0.036). The TG level was significantly higher in the patient group than in the control group (111.5 [37–351] mg/dl vs 90 [34–178] mg/dl; P < 0.001). There was no significant correlation between biochemical parameters and apelin levels in either group (P > 0.05). A significant negative correlation was evident between the apelin level and the THI (r = –0.460, P = 0.003; Figure 2). It was determined that THI decreased when apelin level increased.

**Table:** The apelin levels and biochemical parameters of the patient and control groups.

	Control (n: 40)		Patient (n: 40)		P	Mean ± SD	Median(min–max)	Mean ± SD	Median(min–max)
	Apelin	122.7 ± 44.6	122.6 (521–236)	95.5 ± 31.1		96.7 (34.5–152)	0.002a
Glucose	93.6 ± 8	91.5 (83–118)	92.6 ± 9.3	90 (75–111)	0.671b
HbA1c	5.3 ± 0.3	5.2 (4.8–5.9)	5.4 ± 0.5	5.2 (4.6–6.4)	0.539b
BUN	12.6 ± 3.4	12 (8–23)	13.9 ± 3.5	14 (8–23)	0.097a
Creatinine	0.9 ± 0.1	0.9 (0.7–1.3)	0.9 ± 0.2	0.9 (0.6–1.4)	0.443b
ALT	16.8 ± 5.9	15 (8–35)	17.2 ± 6.1	16 (11–40)	0.746b
AST	18.5 ± 4.7	18 (12–35)	19.4 ± 3.7	18.5 (14–30)	0.147b
cholesterol	189.1 ± 21.8	197.5 (140–233)	187 ± 35.6	184 (125–277)	0.620b
HDL	58.9 ± 11.6	58.5 (30–91)	54 ± 8.8	53 (41–76)	0.036a
LDL	122.5 ± 25.4	114 (80–200)	125.6 ± 30.5	125.5 (66–200)	0.336b
TG	95.2 ± 30.4	90 (34–178)	130.2 ± 54.9	111.5 (37–351)	<0.001b
Hemoglobin	14 ± 1.2	13.7 (12.1–17.4)	13.8 ± 1.4	13.5 (10.1–16.9)	0.350b

## 4. Discussion

Apelin-13 is the active isoform of apelin and binds to APJ (7). In humans, the apelin plasma level is 89.8 ± 5.3 pg/mL (9) and the circulatory halflife approximately 8 min (10). However, Kawamata et al. found that the plasma apelin concentration was lower than that of tissues, suggesting that apelin might be both a circulatory endocrine factor and a paracrine neurotransmitter (11). 

Apelin is a ligand of the G-protein binding receptor APJ, triggering various biological effects. Apelin/APJ signaling plays recognized roles during vasodilation of healthy endothelium, and also during vasoconstriction after vascular endothelial damage (12). The APJ system mediates oxidative stress in vascular tissue (13). Tatemato et al. reported that apelin regulated endothelial nitric oxide (NO) synthesis, increasing NO production. Apelin strongly vasodilated vessels and exerted positive inotropic effects on the heart (14). Yanga et al. showed that, during cerebral ischemia/reperfusion, both apelin-13 and -36 protected neurons from injury caused by neuroinflammation (15). 

Apelin heals ischemic strokes by inhibiting cell death and increasing angiogenesis. Oxidative stress associated with ischemia/reperfusion injury is reduced by increases in the activities of antioxidant enzymes such as superoxide dismutase, catalase, and kidney glutathione peroxidase (16). In an animal study, Chen et al. found that intranasal apelin was a useful stroke treatment (17). The apelin/APJ system regulated insulin sensitivity, gastrointestinal function, angiogenesis, and immune function, enhancing cell migration and proliferation (13). It has been shown that the apelin/APJ system plays various roles in the physiology and pathophysiology of many organs, including regulation of blood pressure, cardiac contractility, angiogenesis, metabolic balance, and cell proliferation, inflammation, or apoptosis. Oxidative stress is considered one of the molecular factors in the pathogenesis of tinnitus (18,19). However, the role of apelin/the APJ system as an oxidative stress mediator in tinnitus is still unknown. The aim of this study was to investigate the apelin mediator as a new oxidative stress parameter in tinnitus patients.

Free radicals damage DNA, proteins, membranes, and intracellular functions, playing roles in the development of atherosclerosis, inflammation, and vascular damage. Neri et al. reported that the plasma levels of oxidative stress markers were significantly elevated in patients with idiopathic tinnitus (18). Oxidative stress increased free radical levels in the labyrinth and the auditory and vestibular pathways, triggering apoptosis of auditory neurons and ciliary cells. Injection into the semicircular canals of materials countering oxidative stress protected the endolymph, neuromediator expression, and bioelectric activity (18,19,20). 

Antioxidants such as N-acetyl cysteine and magnesium prevent oxidative stress, which plays a critical role in the pathogenesis of noise-induced hearing loss and tinnitus preventing apoptosis and cellular necrosis (20). We found that as tinnitus severity increased, the apelin level decreased. Apelin may inhibit the oxidative stress associated with the pathophysiology of idiopathic tinnitus. To the best of our knowledge, this is the first study to explore the relationship between plasma apelin level and tinnitus. 

## 5. Conclusion

This is the first pilot study to explore the relationship between the plasma apelin level and tinnitus. As tinnitus severity increased, the apelin level fell. Thus, apelin may be an independent risk factor in the pathophysiology of idiopathic tinnitus, and may be prescribed during follow-up to reduce oxidative stress in the future. The apelin/apelin receptor (APJ) system may serve as a new therapeutic target in patients with inflammatory diseases associated with oxidative stress, which plays a role in the pathogenesis of inner ear ototoxic lesions. Antioxidants protect reactive cochlear cells. Further clinical studies on the effects of the apelin/APJ system, the role played by oxidative stress, and the effects of antioxidants in patients with inflammatory diseases are required.

## Acknowledgment

This research was funded by the University of Health Sciences Antalya Training and Research Hospital.
